# Crosstalk Between Nitric Oxide and Endocannabinoid Signaling Pathways in Normal and Pathological Placentation

**DOI:** 10.3389/fphys.2018.01699

**Published:** 2018-12-04

**Authors:** Cyntia E. Abán, Paula L. Accialini, Tomás Etcheverry, Gustavo F. Leguizamón, Nora A. Martinez, Mariana G. Farina

**Affiliations:** ^1^Laboratorio de Investigación Aplicada a las Neurociencias (LIAN), FLENI – CONICET, Belén de Escobar, Argentina; ^2^Laboratorio de Fisiopatología Placentaria, CEFyBO-UBA-CONICET, Buenos Aires, Argentina; ^3^Unidad de Embarazo de Alto Riesgo, CEMIC, Buenos Aires, Argentina; ^4^Laboratorio de Biología de la Reproducción, IFIBIO-UBA-CONICET, Buenos Aires, Argentina; ^5^Red Iberoamericana de Alteraciones Vasculares Asociadas a Trastornos del Embarazo (RIVA-TREM), Buenos Aires, Argentina

**Keywords:** placenta, endocannabinoids, nitric oxide, preeclampsia, endothelial disfunction, anandamide

## Abstract

Endocannabinoids are a group of endogenous lipid mediators that act as ligands of cannabinoid and vanilloid receptors, activating multiple signal transduction pathways. Together with enzymes responsible for their synthesis and degradation, these compounds constitute the endocannabinoid system (ECS), which is involved in different physiological processes in reproduction. The placenta, which is essential for the success of gestation and optimal fetal growth, undergoes constant tissue remodeling. ECS members are expressed in trophoblast cells, and current evidence suggests that this system is involved in placental development, apoptosis, and syncytialization. Impairment of endocannabinoid signaling has been associated with several pathological conditions such as intrauterine growth restriction and preeclampsia. Both clinical entities are characterized by dysregulation on vascular perfusion where nitrergic system performs a pivotal role. Nitric oxide (NO) is a potent local vasodepressor that exerts a critical role in the regulation of hemodynamic flow, contributing to the maintenance of low vascular resistance in the feto-placental circulation. NO production could be affected by different factors and growing evidence suggests that the endocannabinoid mediators may regulate nitrergic signaling. Herein, we review emerging knowledge supporting ECS-mediated regulation of NO production in normal placentation. Finally, we discuss how alterations in these systems could affect homoeostasis and contribute to the occurrence of placental-mediated pregnancy complications. Given the impact on women and perinatal heath, we will focus on current knowledge regarding the effects of ECS on nitrergic system in normal and pathological placentation.

## Introduction

The placenta is a specialized transient organ essential for embryo growth and survival. In order to supply the metabolic demands of the developing fetus, this tissue performs numerous physiological functions such as gas exchange and efficient nutrient transfer. These events are crucial for the correct development of the feto-placental unit.

The placenta is an organ devoid of nerves; hence communication between mother and fetus takes place through blood-borne as well as locally produced substances. The syncytiotrophoblast (STB) is the main structural and functional epithelial layer that produces a variety of hormones such as human chorionic gonadotropin (hCG), placental lactogen, estrogen, progesterone, aldosterone, cortisol, placental growth hormone, among others. It can also release a large number of growth factors, cytokines, chemokines, and vasoactive compounds that synchronize placental blood flow, which is of outmost importance during gestation for fetal development (Gude et al., 2004).

Successful pregnancy is coordinated by a complex interplay of maternal, placental, and fetal endocrine signals. Inadequate migration of trophoblast cells and deficient remodeling of uterine spiral arterial walls lead to a reduction of placental blood flow and cause placental ischemia/hypoxia. In this context, vasoactive factors such as inflammatory cytokines, reactive oxygen species, hypoxia-inducible factors (HIFs), and anti-angiogenic factors are the major modulators of the systemic vascular endotheliosis. Both abnormalities in placental formation and function are often associated with human pregnancy complications such as intrauterine growth restriction (IUGR) and preeclampsia (PE).

PE is one of the leading causes of maternal and perinatal morbidity and mortality. In fact, it is the first direct cause of maternal death in Latin America (Giachini et al., 2017). This condition is characterized by hypertension (≥140/90 mmHg) associated to proteinuria (≥0.3 g/24 h) or thrombocytopenia (platelet count < 100.00/μL), liver dysfunction, new onset renal failure (Serum creatinine > 1.1 mg/dL), neurologic symptoms, or pulmonary edema (Brennan et al., 2014).

Endothelial dysfunction is one of the earliest manifestations of PE. To date, the pathogenesis of PE is complex and not well-understood, but it is accepted that an inappropriate remodeling of spiral uterine arteries leads to restricted supply of oxygen and nutrients to the placenta (Li et al., 2015). Vascular endotheliosis associated to PE can lead to a deregulation in the levels of vasodilator factors such as nitric oxide (NO). This altered environment causes placental ischemia and subsequent secretion of placental pro-inflammatory and anti-angiogenic factors into the maternal circulation such as soluble fms-like tyrosine kinase-1 (sFlt-1) and soluble endoglin (sEng), among others (Karumanchi, 2016). Furthermore, evidence of negative correlation between of circulating sFlt-1 and sEng on NO production has been reported in human samples (Sandrim et al., 2008) as well as in animal models of PE (Zhu et al., 2016). However, there are controversies among different studies that measure both circulating levels and urinary excretion of NO in normal and pathological conditions like PE (Ranta et al., 1999; Choi et al., 2002; López-Jaramillo et al., 2008). The discrepancy in the results could be due to different dietary intake of nitrites and nitrates or pharmacological treatments that are given to patients. In this regard, it should be noted that nifedipine, an antagonist of calcium channel widely used for hypertension treatment in preeclamptic patients, may alter NO levels (Berkels et al., 1994; Boccardo et al., 1996).

Furthermore, a number of reports also showed differences in the expression and activity of endothelial NO Synthase (eNOS) between normal and unhealthy pregnancies. (Myatt et al., 1997; Kim et al., 2006; Smith-Jackson et al., 2015; Motta-Mejia et al., 2017).

Distribution and activity of eNOS are regulated by different mechanisms. Trafficking between caveolar and non-caveolar compartments, protein–protein interaction, and phosphorylation are involved in the modulation and/or release of NO (Liaudet et al., 2000; Powe et al., 2011). Therefore, there is an extending interest in determining the specific cellular pathways that modulate the nitrergic signaling. Growing evidence indicates that the endocannabinoid system (ECS) is able to regulate the formation and/or release of NO (Lipina and Hundal, 2017).

The ECS is expressed in human placenta (Park et al., 2003; Aban et al., 2013; Costa et al., 2013) and previous results demonstrate that endogenous cannabinoids (ECs) could modulate NO production acting on different molecular targets (Poblete et al., 2005; Carney et al., 2009; Oddi et al., 2012; Krishnan and Chatterjee, 2015).

Herein, we discuss evidence that supports the role of these endogenous bioactive lipids in the regulation of NO signaling in healthy and pathological pregnancies.

## Role of Nitric Oxide in the Placenta

Throughout gestation significant circulatory adaptations occur that includes an increase in maternal blood volume and vasodilatation to maintain the fetal demands of oxygen and nutrients. Maternal uterine vascular remodeling is essential for normal fetal growth and NO plays a crucial role in this process (Myatt, 1992; Possomato-Vieira and Khalil, 2016).

Over the course of gestation the action of NO seems to support a low vascular resistance in the feto-placental circulation (Amit et al., 1998), maintain a vasodilator state of placental vessels, and attenuate the effects of vasoconstrictors (Myatt et al., 1992) being the main contributor to the regulation of physiological hemodynamic flow.

Nitric oxide is a potent gaseous mediator produced in different organs, including placenta (Farina et al., 2001; Shaamash et al., 2001; Cella et al., 2008; Aban et al., 2013).

During the third trimester, the growing fetus significantly enhances the metabolic demands on the placenta. Changes in vascular resistance allow the placenta to support fetal development and wellbeing. In this remodeling of placental blood-flow, both maternal and conceptus eNOS increase uterine arterial blood flow in normal pregnancy (Kulandavelu et al., 2012), and attenuation in its action may reduce placental perfusion and lead to an altered feto-placental signaling.

Nitric Oxide acts in multiple pathways. It diffuses into vascular smooth muscle cells, attaches to the receptor soluble guanylyl cyclase (sGC), and catalyzes the formation of cyclic guanosinemonophosphate (cGMP), resulting in vasodilation. Simultaneously, NO prevents the production and action of both endothelium-derived contracting factors and endothelin-1, thus reducing the vasoconstrictor effect. Additionally, NO inhibits platelet aggregation and adherence to endothelial surfaces (Ignarro, 1990).

**FIGURE 1:**
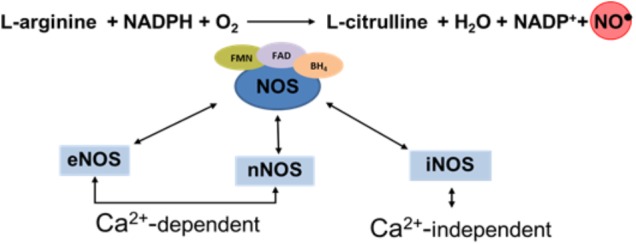
Schematic diagram illustration the synthesis of NO.

Nitric oxide and L-citrulline are produced from L-arginine through a reaction catalyzed by a family of calcium-calmodulin-dependent enzymes called NO synthases (NOS): Three major NOS isoforms have been identified: neuronal (nNOS or NOS1), inducible, (iNOS or NOS2), and endothelial (eNOS or NOS3). The nNOS and eNOS isoforms are frequently expressed constitutively and their activities are regulated by calcium availability. On the other hand, iNOS is independent of the intracellular calcium concentration and generates a high flow of NO. The tree isoforms of NOS employ flavin adenine dinucleotide (FAD), flavin mononucleotide (FMN), and (6R)-5,6,7,8-tetrahydro-L-biopterin (BH_4_) as cofactors of the isozymes (Förstermann and Sessa, 2011) (Figure 1).

These enzymes are present in many cell types and tissues such as endothelium, nerves, immune cells, and placenta. In a normal pregnancy, eNOS is the most relevant member of this family and is the key enzyme when considering the production of NO (Moncada and Higgs, 2006).

In the human placenta, the eNOS isoform is expressed in the STBs and vascular endothelium (Kakui et al., 2003; Schiessl et al., 2005). Interestingly, extravillous trophoblast also produces NO while invading the maternal uterine spiral arteries but significantly higher NOS activity was found in the villous trophoblast. Ca^2+^-dependent NOS activity was also identified in human term placentas, but it is substantially lower respect to samples from early placentas (Al-Hijji et al., 2003). On the other hand, iNOS is expressed in Hofbauer cells of the villous stroma (Myatt et al., 1997).

The placenta lacks of innervation, thus its vascular tone is modulated principally by local factors. In this context, the production of NO is essential for the development of normal placental endothelium, and promotes endovascular invasion by the cytotrophoblast (Zhou et al., 1997). These cells produce NO which acts on arterial walls to create a low-resistance, high-caliber uteroplacental unit (Noris et al., 2005).

The NO production is regulated by many molecules such as vascular endothelial growth factor (VEGF) and placental growth factor (PlGF). Both induce arterial vasodilation by increasing the endothelial calcium signaling, resulting in the release of endothelial NO. Additionally, endothelial shear stress produced by flowing blood stimulates endothelial NO release through a number of pathways, which involve opening of cation channels like TRPV1, TRPV4, among others (Vanhoutte et al., 2016). Protein–protein interactions represent another important mechanism for eNOS regulation. In this context, eNOS can interact with a variety of proteins such as calmodulin or caveolin resulting in an increase or a decrease in eNOS activity (Su, 2014).

## The Endocannabinoid System

Endocannabinoids are an emerging group of lipid-signaling molecules that include amides, esters and ethers of long-chain polyunsaturated fatty acids.

Endocannabinoids are produced on demand by cleavage of membrane phospholipids mainly through two-step reaction catalyzed by *N*-acyltransferase (NAT) and *N*-acylphospha tidylethanolamine-phospholipase D (NAPE-PLD) in the pathway of Anandamide (*N*-arachidonoylethanolamine; AEA) synthesis; and phospholipase C (PLC) and diacylglycerol lipases (DAGL) in the case of 2-arachidonoylglycerol (2-AG).

Both lipid mediators (AEA and 2-AG) are the main endogenous ligands of the cannabinoid receptors (CB1 and CB2) (Howlett et al., 2002). These receptors belong to the family of G-proteins coupled receptors (GPCRs) and activate multiple signaling pathways (Pertwee, 2006). In addition, these bioactive lipids can stimulate other membrane proteins such as the orphan G protein-coupled receptor 55 (GPR55) (Sharir et al., 2012; Gasperi et al., 2013), or the intracellular receptor peroxisome proliferator-activated receptors (PPAR). Additionally, other ECs such as oleoylethanolamide and palmitoylethanolamide can also bind to the peroxisome proliferator-activated receptor gamma (PPAR-γ) regulating food intake, lipid metabolism, and inflammatory processes (O’sullivan, 2007; Pistis and Melis, 2010). Moreover, the endocannabinoid AEA can bind to a non-selective cation channel, the transient receptor potential vanilloid 1 (TRPV-1), acting as an endovanilloid (Cella et al., 2008; Marzo and Petrocellis, 2010).

The action of AEA and 2-AG cease by enzymatic hydrolysis mediated by fatty acid amide hydrolase (FAAH) (McKinney and Cravatt, 2005; Fezza et al., 2008) and monoacylglycerol lipase (MAGL), respectively (Dinh et al., 2002).

In addition, there are other enzymes that constitute alternative biosynthetic and degradative pathways for this lipid mediators (Kozak et al., 2002; Pacher and Kunos, 2013).

Altogether, these enzymes and proteins involved in the production and signaling of endocannabinoids, along with these lipid ligands, constitute a complex system called ECS.

### Endocannabinoid System in the Placenta

In the last years, enzymes that participate in AEA and 2-AG synthesis and release have been identified in human placenta (Aban et al., 2013; Costa et al., 2013), but until now only AEA levels were measured in this tissue (Marczylo et al., 2010).

The identification of the different components of the ECS in the placenta promoted the study of ECs in relevant physiological processes such as proliferation, differentiation, apoptosis, and proteins biosynthesis, as well as in the transport of nutrients, oxygen, electrolytes, and other substances to the fetus. The results observed in these studies were extensively reviewed by Costa (2016) and the relevance of the ECS in trophoblast biology is summarized in Table 1.

**Table 1:** Processes modulated by AEA and 2-AG in the human trophoblast.

Proliferation			

AEA	↓ mainly through CB2	BeWo	Habayeb et al., 2008a; Costa et al., 2014a, 2015b
2-AG	↓ mainly through CB2	BeWo	

**Cell death**			

AEA	↑ through CB1	hST	Aban et al., 2013
	↑ through TRPV-1	hCT	Costa et al., 2015b
	↑ mainly through CB2	BeWo	Habayeb et al., 2008a; Costa et al., 2014a
2-AG	↑ mainly through CB2	BeWo	

**Syncytialization**			

AEA	? morphological differentiation	hCT	Costa et al., 2014b
	- biochemical differentiation	hCT	
2-AG	↓ morphological differentiation through CB1 and CB2	hCT	Costa et al., 2015c
	↓ biochemical differentiation through CB1 and CB2	hCT	

**Migration and invasion**			

CB1-/-	↓ invasion	TSC	Sun et al., 2010
**Protein biosynthesis**			
AEA	↓ ecto-pALP activity, hCG secretion and aromatase expression through CB receptors	hST	Costa et al., 2015b
	- PAPP-A mRNA levels	hST	Costa et al., 2016
2-AG	↑ 3β-HSD mRNA levels through CB receptors	hST	
	- PAPP-A mRNA levels	hST	

**Transport**			

AEA	↓ K+ channel 1 (TASK-1)	hST	Bai et al., 2006; Wareing et al., 2006
	↓ folic acid transportation, acute treatment. Not mediated by CB receptors	BeWo	Araujo et al., 2009
	↑ folic acid transportation, chronic exposure. Not mediated by CB receptors	BeWo	


*Increase (↑), decrease (↓), no effect (–); AEA, anandamide; 2-AG, 2-arachidonoylglycerol; CB1, cannabinoid receptor 1; CB2, cannabinoid receptor 2; TRPV1, transient receptor potential vanilloid 1; hCT, human cytotrophoblast; hST, human syncytiotrophoblast; TSC, trophoblast stem cells; PAPP-A, Pregnancy-associated plasma protein A; 3 β-HSD, 3 β-hydroxysteroid dehydrogenase; ecto-pALP, placental alkaline phosphatase; hCG, human chorionic gonadotropin.*

In addition to the effects of ECs, phytocannabinoids such as delta-9-tetrahydrocannabinol (THC), the main psychoactive compound of marijuana, may affect the dynamics of placental development (Ortigosa et al., 2012; Costa et al., 2015a; Metz and Stickrath, 2015). In fact, it has been shown that THC can promote beneficial or detrimental effects on trophoblast cell viability and also impair morphological differentiation (Costa et al., 2015a). Additionally, chronic exposure to THC may affect the maternal–fetal transference of micronutrient (Araujo et al., 2009). For all the above mentioned, cannabis consumption during pregnancy may have serious alterations in human placentation causing negative pregnancy outcomes such as preterm birth (Dekker et al., 2012) and fetal growth restriction (El Marroun et al., 2009).

A similar mechanism seems to occur when high levels of endocannabinoids are detected during pregnancy. According to this, reports have shown that high plasma levels of AEA seriously interfere in the progression of pregnancy (Habayeb et al., 2008b; Taylor et al., 2011). In agreement with this observation, previous results from our laboratory demonstrated that NAPE-PLD and FAAH expression were impaired in PE placentas. Both proteins were mainly located in the apical membrane of STB in normal placentas although weak staining for FAAH was detected in some villi from PE tissues. Furthermore, high levels of FAAH activity were measured in normal tissues, but a lower activity of this metabolizing enzyme was detected in preeclamptic tissues (Aban et al., 2013). These findings suggest that pathological conditions may expose the fetus to unhealthy levels of the endocannabinoid, disturbing fetal development, and leading to neurophysiological abnormalities (Grant et al., 2017). However, the precise mechanisms by which the principal enzymes involved in the synthesis and degradation of AEA are deregulated in preeclamptic placentas are still unknown.

Other works have described alterations of several components of the ECS in normal and pathological human placentas. Acone et al. (2009) compared samples obtained from women undergoing elective cesarean section (non-laboring group) and women having a normal spontaneous delivery (laboring group) at term (Acone et al., 2009). Interestingly, CB1 expression was detected but FAAH protein was absent in the analyzed samples. On the other hand, Fügedi et al. (2014) observed higher levels of CB1 protein in the STB layer, as well as in the endothelial cells from preeclamptic placental tissue, although they did not find significant differences in CB2 and FAAH expression between preeclamptic and normal placental tissues (Fügedi et al., 2014).

It is worth to note the discrepancy in the results observed by different research groups on the altered expression of the ECS components, even when the same type of samples was analyzed. Such differences could be attributed to ethnicity, severity of the disease and/or differences in methodological procedures (e.g., sample processing, antibodies utilized). These disagreements must be analyzed and requires further elucidation.

## Crosstalk Between ECS and NO in Reproductive Tissues

Our understanding on the interaction between the ECS and nitrergic system has been enriched by several studies that demonstrated a strong influence of ECS on NO production. This regulation is mediated by endocannabinoids like AEA or 2-AG which exert stimulatory or inhibitory effects depending on tissue context, cell type, and/or activation of specific receptors (cannabinoid receptors or alternative molecular targets). Also, previous reports have provided evidence that a bidirectional modulation exists between the ECS and NO, and this crosstalk is extremely important since alterations in one or both systems would impact on cellular homeostasis or could trigger a pathological condition. A comprehensive review of these interactions is well described in Lipina and Hundal (2017).

Regulation of NO production by the ECS was demonstrated in different biological systems such as neurohypophysis (Luce et al., 2014), retina (Krishnan and Chatterjee, 2015), platelets (Signorello et al., 2011), heart (González et al., 2011), nephron (Mukhopadhyay et al., 2010a,b), and in energy metabolism (Tedesco et al., 2008). Nevertheless, little is known about the ECS-associated interaction with NO during pregnancy. The crosstalk between ECS and NO is relevant in reproductive tissues like bovine epithelial oviduct and spermatozoa (Osycka-Salut et al., 2012), as well as in murine and rat uterus and decidua (Vercelli et al., 2009b; Sordelli et al., 2011). NO is involved in various reproductive events including implantation, regulation of placental blood flow, and myometrial relaxation. However, there are limited reports that explain the mechanisms involved in regulation of ECS on NO production. In murine uterus incubated with lipopolysaccharide (LPS), AEA mediates LPS-induced NO production through activation of both cannabinoid receptors, CB1 and CB2. This lipid mediator increases iNOS expression and pharmacological blockade of CB1 and CB2 inhibit this effect suggesting the participation of both receptors. Moreover, LPS modulates the expression of the enzymes involved in AEA metabolism, producing alterations in AEA levels which results in different types of responses that affect NO production (Vercelli et al., 2009a). A similar mechanism was described in murine decidua, where AEA mediates LPS-induced NO synthesis through activation of both cannabinoid receptors. In this tissue, LPS has a deleterious effect on the implantation sites via CB1 receptor and it is believed that this could be associated to septic abortion (Vercelli et al., 2009b). Furthermore, during the implantation process in rat uterus, AEA modulates NOS activity and NO production on implantation and inter-implantation sites in a specific manner, activating CB1 and/or CB2 depending on the presence or absence of the blastocyst (Sordelli et al., 2011).

The ECS regulates the homeostasis through a wide variety of mechanisms. It facilitates the intracellular communication between different cell types and contributes to maintaining the balance in the body. The placental abnormal expression of the ECS has been associated which serious pregnancy complication such as spontaneous miscarriage (Trabucco et al., 2009) and preterm birth (Sun et al., 2016).

Additionally, it was demonstrated that uterine deregulation of the ECS increases the levels of prostaglandins contributing to the mechanism by which infection causes preterm birth (Bariani et al., 2015). In this animal model, resveratrol administration prevented the changes in the uterine endocannabinoid profiling altered by LPS and diminished iNOS expression and NOS activity evidencing tocolytic effects (Bariani et al., 2017). Additionally, the loss of CB1 receptor has been linked to this pathology (Wang et al., 2008) while others demonstrated that THC has a preventive effect on preterm delivery in a LPS-induced murine model, suggesting the contribution of NO coupling through the CB1 receptor (Asghari-Roodsari et al., 2010).

Endocannabinoids have also been implicated in blood pressure regulation (Pacher et al., 2005). These lipid mediators can cause vasodilation through CB1, TRPV1, and NO-mediated or NO-independent mechanisms (Pacher and Steffens, 2009). Anandamide exerts its vasorelaxant effect on endothelium by upregulating the expression and activity of the inducible NO synthase (NO-mediated pathway) (Randall et al., 2002; Cella et al., 2008). Although there is no direct correlation between AEA serum levels and blood pressure, given these results it is possible to speculate that the decrease in AEA levels observed in preeclamptic pregnant woman (Molvarec et al., 2015) could contribute to their increase in blood pressure, which is a crucial factor characteristic of PE.

In rat placenta, a report from our laboratory demonstrates that AEA exerts a dual effect on NO production depending on which receptor is activated. While activation of TRPV-1 receptor stimulates NO production, the action of AEA on CBs decreases NOS activity, suggesting that AEA acts as a differential fine-tuning regulator of NO during pregnancy (Cella et al., 2008).

In fact, although AEA activates TRPV-1, the concentration required is higher than that needed for CB1 activation (Ross, 2003). On the other hand, an opposite effect is observed in human tissues. Interestingly, in human placenta at term both endogenous and exogenous AEA increase NOS activity through CB1 receptor (Aban et al., 2013). It is important to highlight that the activation of different receptors induces opposite responses, and this effect could be associated to changes in ECS which cause an appropriate AEA “tone”, contributing to trigger one or other type of response. We speculate that the differences observed between rat and human placentas concerning to the effect of AEA on NOS activity may be due to the different gestational times analyzed, activation of different signaling pathways of CBs, and also to the expression of TRPV-1 that changes at the end of pregnancy.

**FIGURE 2:**
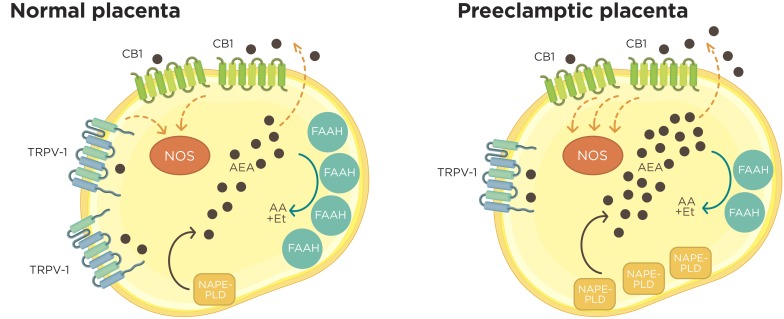
Croostalk between ECS and nitrergic system in normal and preeclamptic placentas. In normal placenta, synthesized AEA from membrane lipids by NAPE-PLD leave to the trophoblast by simple diffusion or by carrier proteins and interact with CB1 receptor. The activation of CB1 by AEA results in the activation of NOS activity. Higher FAAH expression and activity contributes to the maintenance of a low AEA “tone” degrading this lipid to arachidonic acid (AA) and ethanolamine (Et). Inside the trophoblast cells, AEA also could regulate NOS activity through TRPV1 receptor. On the other hand, in preeclamptic placentas, an increased NAPE-PLD expression associated with lower FAAH expression and activity encourage a raise in AEA “tone” that contributes to a higher NO production at least in part through CB1 receptor.

In pathological conditions like PE, a higher basal NOS activity was observed in comparison to healthy normal samples. This observation, together with the altered expression pattern of the ECS metabolic enzymes, could result in higher AEA levels, which positively stimulate NOS activity and NO production (Aban et al., 2013) (Figure 2). Additionally, preliminary results obtained in our laboratory suggest that changes in the expression of some components of the ECS in human laboring placentas at term also modify NOS activity during labor (unpublished data).

Altogether, the results discussed in this review indicate that either the activation or the inhibition of the ECS can alter the production of NO, leading to beneficial or prejudicial biological responses depending on the cell type. Because the ECS and NO signaling are involved in the modulation of relevant aspects of placental physiology such as vasodilatation and placental blood flow, it is crucial for the tissues to keep their levels acutely regulated. Thus, it is expected that a crosstalk between these systems may contribute to the maintenance of the tissue homeostasis.

Given the relevance of the nitrergic signaling and the ECS in the development of placenta, this review may contribute to identify novel targets for the treatment of placental diseases such as PE.

## Summary

In order to understand the functionality of the placenta, we must take into account the complexity of the events that occur in this organ. In this review we have focused and discussed about the importance of ECS and NO in the physiological behavior of normal and pathological placentas. The ECS acts as a regulator of nitrergic system, modulating NO levels. Since NO is the main vasodilator in human placenta implicated in modulation of blood flow, alterations in this mediator may modify placental functions and can be associated to pathological conditions of pregnancy like PE. Herein we summarize recent experimental findings that support the importance of a crosstalk between AEA and NO and the contribution of CB1 signaling in placental development in normal and pathological conditions of pregnancy. Altogether this evidence proposes the ECS as a part of a relevant mechanism of the placenta and may serve as a possible pharmacological target given the relevance of this system in the regulation of NO and, consequently, in placental vascular dysfunction.

## Author Contributions

CA and MF have proposed the topic of this revision and designed the figures. All authors have contributed to information recrutment and write the present version.

## Conflict of Interest Statement

The authors declare that the research was conducted in the absence of any commercial or financial relationships that could be construed as a potential conflict of interest.
